# Current Perspectives on Pharmacological and Non-Pharmacological Interventions for the Inflammatory Mechanism of Unipolar Depression

**DOI:** 10.3390/brainsci12101403

**Published:** 2022-10-18

**Authors:** Ioana-Alexandra Dogaru, Maria Gabriela Puiu, Mirela Manea, Vlad Dionisie

**Affiliations:** 1 Faculty of Medicine, “Carol Davila” University of Medicine and Pharmacy, 020021 Bucharest, Romania; 2 Department of Psychiatry and Psychology, “Carol Davila” University of Medicine and Pharmacy, 020021 Bucharest, Romania

**Keywords:** depression, anti-inflammatory, NSAID, cytokine inhibitor, statins, minocycline, N-acetyl cysteine, omega-3 polyunsaturated fatty acids, electroconvulsive therapy, physical therapy

## Abstract

Since depression remains a major public health issue there is a constant need for new and more efficient therapeutic strategies based on the mechanisms involved in the aetiology of depression. Thus, the pathogenic link between depression and inflammation is considered to play a potential key role in the development of such therapies. This review summarizes the results of various pharmacological (non-steroidal anti-inflammatory drugs, aspirin, cyclooxygenase inhibitors, cytokine inhibitors, corticosteroids, statins, minocycline, N-acetyl cysteine, omega-3 fatty acids and probiotics) and non-pharmacological interventions (electroconvulsive therapy, physical exercise and psychological therapy) and outlines their efficacy and discusses potential challenges. Both conventional and non-conventional anti-inflammatory drugs showed promising results according to the specific group of patients. The pre-existing pro-inflammatory status was, in most cases, a predictor for clinical efficacy and, in some cases, a correlation between clinical improvement and changes in various biomarkers was found. Some of the non-pharmacological interventions (physical exercise and electroconvulsive therapy) have also showed beneficial effects for depressive patients with elevated inflammatory markers. Treatments with anti-inflammatory action may improve clinical outcomes in depression, at least for some categories of patients, thus opening the way for a future personalised approach to patients with unipolar depression regarding the inflammation-related mechanism.

## 1. Introduction

Major depressive disorder (MDD), often referred to as unipolar depression, is an important public health issue nowadays, affecting around 280 million people worldwide, which corresponds to approximately 3.8% of the population [1]. Patients with MDD experience only major depressive episodes, while patients with bipolar disorder exhibit mood fluctuations which encompass depressive episodes, known as bipolar depression in this case, and episodes of mania or hypomania [2]. MDD is characterised by symptoms such as depressed mood, anhedonia, appetite and sleep dysfunctions, psychomotor agitation or retardation, fatigue, feelings of worthlessness, thinking or concentration problems and suicidal ideation [3]. Its impact on health can be dramatic, as it increases the risk of cardiovascular disease, stroke, diabetes and obesity [4], and suicide is one of the leading causes of death, especially in the 15–29 age group [1]. Moreover, treatment-resistant depression is an important challenge in clinical practice since 10–30% of patients are refractory to several standard antidepressant medications and have a decreased quality of life [5]. Considering the various implications of this disorder, which far exceed those listed here, the constant need for developing new and efficacious therapeutic strategies seems perfectly justified.

Current medications approved for treating MDD are selective serotonin reuptake inhibitors—SSRIs, serotonin and norepinephrine reuptake inhibitors—SNRIs, tricyclic antidepressants—TCAs, monoamine oxidase inhibitors—MAOIs, N-Methyl-D- aspartate (NMDA) receptor antagonists, serotonin modulators and atypical antidepressants. They are thought to exert their actions mainly by increasing the available synaptic serotonin and/or norepinephrine [6]. This mechanism is based on the monoaminergic theory of depression, which states that a decrease in serotonin, norepinephrine and dopamine is responsible for this pathology. Chronologically, it is the first proposed theory (hence the inherent limitations of these drugs) [7]. However, with immunology as a rapidly emerging field and inflammation incriminated as an underlying cause of many diseases [8], there is growing evidence for a putative link between inflammation and depression. This finding holds promise for new possible approaches in addressing this challenging disorder [9]. Interestingly, SSRIs and SNRIs were proved to have an anti-inflammatory role in the central nervous system (CNS) which may play a part in the antidepressant effect [10].

Depression was shown to be associated with morphofunctional changes at the level of various brain areas, such as the frontal and parietal cortex, the hippocampus, the thalamus or the striatum [11]. These changes represent the substrate for the cognitive and behavioural impairments seen in this pathology. For example, striatal gray matter alterations are correlated with suicidality [12], whereas dysfunctions of the prefrontal cortex–amygdala–hippocampus circuitry possibly connected with neurovisceral structures lead to abnormal fear conditioning [13,14]. Regarding inflammatory depression, structural and functional changes in the aforementioned brain regions were identified in the context of elevated peripheral inflammatory biomarkers [15]. For instance, increased C-reactive protein (CRP) is associated with a dysfunctional corticostriatal reward circuit—a key component of treatment-resistant depression [16]. At the molecular level, neuronal impairment could be explained by the direct neurotoxic effect of inflammatory cytokines. Moreover, cytokines appear to stimulate the tryptophan-kynurenine-quinolonic acid pathway, inducing excitotoxicity [15]. Indeed, there is increasing evidence for kynurenine pathway activation in MDD patients [17].

Mechanistic explanations for inflammation-associated depression relate to the action of cytokines on basal ganglia [18] and several polymorphisms in cytokine genes were associated with depression and response to antidepressants [19]. Sometimes, inflammatory depression is even discussed as an entirely particular subtype of depression and seems to be correlated with the prevalence of certain symptoms, such as hypersomnia, fatigue, or increased appetite. These symptoms also belong to the “atypical” subtype, which occurs in approximately 15–30% of patients [4]. Atypical depression was shown to be connected with inflammation, although some results were not consistent [20]. Of note, obesity and metabolic syndrome (inflammation-related conditions) are common findings in patients presenting with this subtype [21]. Fatigue might also be the expression of “sickness behaviour”. This energy-conserving adaptive response to infections and other inflammation-inducing situations displays similarities with depression [22]. As opposed to the atypical subtype, melancholic depression features reduced pro-inflammatory cytokines, except for exacerbations [23]. Finally, inflammation was also associated with treatment-resistant depression [24]. All these findings underline the heterogeneous nature of depression and support the need for an individualised approach to patients exhibiting different clinical pictures.

Importantly, numerous authors have suggested that treatment with anti-inflammatory effects should be administered only to a subgroup of MDD patients. These patients should possess clear signs of inflammation (e.g., increased level of plasma cytokines/acute phase proteins, presence of diseases with a strong inflammatory component, such as psoriasis or osteoarthritis) [25,26]. Moreover, the opposite might lead to detrimental outcomes, as reported in patients lacking such changes in biomarkers who were treated with tumour necrosis factor (TNF) inhibitors [26]. Even if the relationship between depression and inflammation appears to be intricate and more preclinical data is needed to elucidate it, clinical trials of therapies displaying anti-inflammatory action have shown encouraging results over time.

The present review aims to explore various pharmacological (i.e., non-steroidal anti-inflammatory drugs—NSAIDs, cytokine inhibitors, corticosteroids, statins, minocycline, N-acetyl cysteine—NAC, omega-3 fatty acids, probiotics) and non-pharmacological interventions (i.e., electroconvulsive therapy—ECT, physical exercise and psychological therapy). Their efficacy and potential challenges are discussed, providing an enlarged, although not exhaustive, perspective on the specific aspects of the anti-inflammatory treatment in unipolar depression. Also, this paper aims to set the scene for a future personalised approach to patients with unipolar depression with respect to the inflammation-related mechanism.

## 2. Methodology

Multiple advanced searches were performed in the PubMed, Web of Science and Scopus databases during November 2021–August 2022 using the terms “NSAID”, “aspirin”, “COX-2 inhibitor”, “celecoxib”, “cytokine inhibitor”, “infliximab”, “etanercept”, “corticosteroids”, “statins”, “minocycline”, “N-acetyl cysteine”, “omega-3 fatty acids”, “probiotics”, “physical exercise”, “ECT”, “electroconvulsive therapy”, “psychological therapy”, cross-referenced with “depression”, “major depression” and “MDD”. The inclusion criteria considered were: articles written in English; full-text available; randomised controlled trial (RCT), meta-analysis and systematic review; the articles investigated the antidepressant effect of the interventions mentioned above. The exclusion criteria considered were: articles written in other language than English; studies regarding bipolar depression; other clinical studies than RCTs, preclinical studies, narrative reviews, letters to the editor, book chapters, case reports, conference presentations, interviews; full-text was not available. Moreover, for non-pharmacological interventions (i.e., ECT, physical exercise, and psychological therapy), studies were included if they also explored inflammatory markers in relation to the antidepressant action in order to be relevant to the topic under discussion. This review mainly focuses on the discussion of RCTs since they have the highest scientific level of evidence. However, in the case of aspirin, statins, NAC, physical exercise, ECT and psychotherapy there was a scarce number of available RCTs. Therefore, for these interventions, other types of clinical studies were included (i.e., open-label, cohort, naturalistic or register-based studies) in order to be able to provide a better understanding of their effects. References from narrative reviews, systematic reviews and meta-analyses were also carefully examined to identify other studies that met the inclusion and exclusion criteria. In the end, a number of 153 articles were selected and divided as it follows: 24 for NSAIDs, 13 for cytokine inhibitors, 6 for corticosteroids, 12 for statins, 6 for minocycline, 4 for NAC, 37 for omega-3 fatty acids, 16 for probiotics, 9 for physical exercise, 13 for ECT, 13 for psychotherapy.

## 3. Conventional Anti-Inflammatory Drugs

### 3.1. Non-Steroidal Anti-Inflammatory Drugs (NSAIDs)

Perhaps some of the most used and studied anti-inflammatory treatment options are NSAIDs. They act by inhibiting the enzymes cyclooxygenase (COX)-1 and COX-2, which promote inflammatory mediators [25]. NSAIDs divide into non-selective and selective (only inhibiting COX-2). The first category includes drugs such as aspirin, diclofenac, ibuprofen, indomethacin, meloxicam, or naproxen, whereas celecoxib and etoricoxib are notable members of the second one [27].

Among non-selective NSAIDs, aspirin was shown to be a potentially successful add-on. It even decreased latency to SSRIs response in a pilot open-label study where 52.4% of the patients not previously responding to SSRI therapy showed clinically relevant improvement, mostly from the first week [28]. Moreover, treatment with aspirin was linked to a lower depression rate in the first year after a primary tumour diagnosis. This result was not achieved by other NSAIDs [29]. Unfortunately, there is only a small number of RCTs, with conflicting results. For instance, in a relatively recent study, the co-administration of aspirin and citalopram had to be interrupted, due to severe adverse reactions (anxiety, akathisia and even suicidal behaviour) (Table 1) [30]. However, we have to consider the very small number of patients included in this study and that these side effects can rather be attributed to citalopram than aspirin. While aspirin as an add-on to sertraline elicited a greater benefit than sertraline-placebo alone [31], another RCT, with escitalopram and duloxetine as concomitant medication, identified a difference only between two subgroups (i.e., duloxetine + aspirin and escitalopram + placebo). This result could also be attributed to higher baseline serum brain-derived neurotrophic factor (BDNF) in the first subgroup (Table 1) [32]. Two major trials from 2020 failed to show the benefit of aspirin in treating young MDD patients or preventing depression in the elderly. Berk et al. investigated the effects of rosuvastatin or aspirin in young people and found no advantage of the two over placebo. Furthermore, at week 12, aspirin was inferior to placebo in improving patients’ quality of life and to rosuvastatin on several parameters, including depression intensity [33]. Concerning a possible prophylactic effect of aspirin, the results of a multicentre, double-blinded RCT did not support a preventive role of low-dose aspirin in depression nor identify any change in depressive symptoms in participants with a history of this disease (Table 1) [34]. A recent meta-analysis and a systematic review attained contradictory conclusions concerning the correlation between aspirin and depression, proving the need for more extensive trials. One of them found a positive association, although infrequent, with a number needed to harm of 103 [35]. The other one demonstrated a link between aspirin and a reduced risk of developing depression [36]. To conclude, there is a clear need for further investigation, but the results are in favour of the add-on type of treatment regimen, especially in clinically depressed individuals. A summary description of the RCTs of NSAIDs in MDD can be found in Table 1.

In turn, naproxen was beneficial in patients with comorbid osteoarthritis and not in older healthy individuals in two randomised clinical trials, proving once again the need for a pre-existing pro-inflammatory status [37,38].

Of the selective COX-2 inhibitors, celecoxib was the most investigated as an adjunctive to antidepressants, but also in monotherapy, leading to promising results. Its effectiveness as an add-on was repeatedly proved by RCTs on patients with depression [39,40], but also with colorectal cancer [41] or with depression and comorbid brucellosis [42]. Monotherapy in patients with active osteoarthritis was also successful, similar to naproxen or ibuprofen, even if the celecoxib dose was lower than in other studies (200 versus 400 mg/day) [42]. The connection between treatment response and immunological biomarkers is supported by the correlation between interleukin (IL)-6 reduction and Hamilton Depression Rating Scale (HAM-D) score reduction [43], together with a tendency towards higher baseline macrophage migration inhibitory factor (MIF) levels in responders [44]. On the other hand, a recent RCT failed to show any benefit of celecoxib added to vortioxetine, even when patients were stratified by high-sensitive C-reactive protein (hsCRP), but it is worthy of note that other biomarkers were not examined [45].

Various concerns are related to the use of NSAIDs in unipolar depression, as it is known that they reduce the multifunctional protein p11 levels. This protein is associated with antidepressant response and is upregulated by SSRIs by means of cytokines [18]. Thus, NSAIDs might be appropriate for enhancing treatment with TCAs or noradrenergic antidepressants, but not SSRIs [46]. Selective COX-2 inhibition promotes nitrosative and oxidative stress, decreases immunomodulator prostaglandin E2 (PGE2), and may engender psychiatric symptoms [29]. NSAIDs are also incriminated for numerous adverse effects, most notably gastrointestinal and cardiovascular [27]. However, a meta-analysis did not identify such effects associated with this particular use in depression (with limitations concerning the duration and the lack of report) [26]. It is hence argued that they might be an advantageous addition particularly in the treatment of patients at low risk of cardiovascular events, except for low-dose aspirin, which is cardioprotective [47]. Finally, a register-based study revealed that aspirin and other NSAIDs decrease the risk for early-onset depression after a first stroke episode, where inflammation is presumed to play a pivotal role. At the same time, they were associated with a high risk of depression one year after the episode, highlighting the multifaceted nature of these drugs [48]. Nonetheless, they might be considered as valuable additions to antidepressant treatment, when accounting for the patient’s characteristics (clinical depression, comorbidities, inflammatory status).

**Table 1:** Summary of randomised controlled trials of NSAIDs in MDD.

Study	Drug and Treatment Regimen	Participants	Duration of Intervention	Immune Parameters	Main Outcomes
Ghanizadeh, 2014 [30]	Aspirin, 160 mg/day (add-on to citalopram, 20 mg/day)	10 adult out-patients with MDD	14 days; discontinued in 8 out of 10 patients	Not measured	Harmful
Zdanowicz, 2017 [32]	Aspirin, 100 mg/day (add-on to escitalopram or duloxetine)	40 individuals with MDD	2 years	Not measured	Not effective (but duloxetine + aspirin superior to escitalopram + placebo)
Sepehrmanesh, 2017 [31]	Aspirin, 2 × 80 mg/day (add-on to sertraline, 50–200 mg/day)	100 patients with MDD	8 weeks	Not measured	Effective
Berk, 2020 (YoDA-A) [33]	Aspirin, 100 mg/day or rosuvastatin 10 mg/day (add-on to treatment as usual)	130 young people (15–25 years) with moderate to severe MDD	12 weeks	Not measured	Not effective
Müller, 2006 [39]	Celecoxib, 400 mg/day (add-on to reboxetine 4–10 mg/day)	40 patients with MDD	6 weeks	Not measured	Effective as add-on
Akhondzadeh, 2009 [40]	Celecoxib, 2 × 200 mg/day (add-on to fluoxetine 40 mg/day)	40 adults with MDD	6 weeks	Not measured	Effective as add-on
Musil, 2011 [49]	Celecoxib, 400 mg/day (add-on to reboxetine 4–10 mg/day)	32 patients with MDD and 20 healthy controls	6 weeks	No difference in MIF, TGF-β and sCD14	Effective as add-on
Abbasi, 2012 [43]	Celecoxib, 2 × 200 mg/day (add-on to sertraline 200 mg/day)	40 patients with MDD	6 weeks	Reduced IL-6	Effective as add-on
Majd, 2015 [50]	Celecoxib, 2 × 100 mg/day (add-on to sertraline, 25, then 50 mg/day)	30 women with MDD (first episode), 18–50 years old	8 weeks	Not measured	Effective after 4 weeks, not significant after 8 weeks
Alamdarsaravi, 2017 [41]	Celecoxib, 400 mg/day (monotherapy)	40 patients with mild to moderate MDD and colorectal cancer	6 weeks	Not measured	Effective
Krause, 2017 [51]	Celecoxib, 400 mg/day (add-on to reboxetine, 4–10 mg/day)	40 patients with MDD and healthy controls	6 weeks	Not measured	Remission in celecoxib group predicted by higher KYN/TRP baseline ratio
Baune, 2021 [45]	Celecoxib, 400 mg/day (add-on to vortioxetine 5–10 mg/day)	119 patients with MDD	6 weeks	Participants were stratified by hsCRP (> or ≤3 mg/L)	Not effective
Simon, 2021 [44]	Celecoxib, 2 × 200 mg/day (add-on to sertraline, 50–150 mg/day)	43 patients with MDD, 18–60 years old	6 weeks	MIF: lower at baseline in placebo remitters than non-remitters, trend for higher baseline levels in celecoxib responders than non-responders; neopterin, TNF-α: no clear pattern	Not effective
Mohammadinejad, 2015 [52]	Celecoxib, 2 × 200 mg/day or diclofenac, 2 × 50 mg/day (monotherapy)	52 patients with MDD and breast cancer	6 weeks	Not measured	Celecoxib more effective than diclofenac

MDD, major depression disorder; MIF, macrophage migration inhibitory factor; TGF-β, Transforming growth factor-β; sCD14, soluble CD14; IL, interleukin; TNF-α, tumour necrosis factor-alpha; KYN, kynurenine; TRP, tryptophan; hsCRP, high-sensitive C reactive protein.

### 3.2. Cytokine Inhibitors

As NSAIDs are often thought to be too “off-target”, cytokine inhibitors, which have a history of inflammatory conditions treatment, may be more suitable for the purpose under discussion. Indeed, they have been studied and proved effective, especially in the presence of such comorbidities [53,54]. A problem that arises is that it is not always clear if the reduction in depressive symptoms is direct or indirect, due to a change in the primary disease’s characteristics. For example, in a study on patients with hidradenitis suppurativa, adalimumab led to a greater improvement in participants with higher baseline pain [55].

Supporting the theory of a separate depression subtype, they were most effective when plasma cytokine levels were increased. For instance, infliximab—a TNF antagonist, surpassed placebo in depression score reduction only in the hsCRP > 5 mg/L group. This effect was enhanced by high baseline TNF together with its soluble receptors. In contrast, placebo was superior in the baseline hsCRP ≤ 5 mg/L group [18]. Conversely, another RCT of infliximab reported an improvement in depressive symptoms which was no longer significant after eliminating the influence of ankylosing spondylitis disease activity and did not correlate with CRP levels [53]. Adalimumab and etanercept, other TNF-α antagonists, improved depression scores in patients with rheumatic (i.e., ankylosing spondylitis), Crohn’s disease, psoriasis or hidradenitis suppurativa and comorbid depressive symptoms [54,55,56,57,58]. Dupilumab, an antagonist of the receptor of IL-4, showed similar results to those of the anti-TNF-α monoclonal antibodies concerning antidepressant efficacy [59,60,61]. A summary description of the RCTs of cytokine inhibitors in patients with MDD or with depressive symptoms and comorbid medical condition can be found in Table 2.

Several less common drugs target specific cytokines whose exact role in depression is not known, but which seem to be, sometimes, even more effective. Such is the case of ixekizumab, an IL-17A inhibitor which, according to data from a recent RCT, improved depressive symptoms in patients with psoriasis, whereas TNF-α inhibitor etanercept did not, suggesting a particular role of IL-17A in the CNS [62]. Another example, guselkumab, an IL-23 inhibitor, was investigated in psoriasis patients and was proved superior to adalimumab, maintaining its outcome on depression even after adjustment for the effects related to disease activity [63].

There remains the threat of potentially serious side effects, such as infections, which were not identified by the previously mentioned meta-analysis conducted by Köhler et al. [26], although in the context of a small number of studies. As outlined by the existing data, cytokine inhibitors possess a more targeted effect on depression-related inflammation than those previously discussed and show some promise in alleviating depressive symptoms in specific groups, i.e., patients with pre-existing inflammatory comorbidities, but more research is needed to reveal the exact effect in MDD without comorbid medical conditions.

**Table 2:** Summary of randomised controlled trials of cytokine inhibitors in patients with MDD or with depressive symptoms and comorbid medical condition.

Study	Drug and Treatment Regimen	Participants	Duration of Intervention	Immune Parameters	Main Outcomes
Raison, 2013 [18]	Infliximab (3 infusions of 5 mg/kg at baseline and weeks 2 and 6) (monotherapy or add-on to treatment as usual)	60 patients with treatment-resistant MDD	12 weeks	Greater decrease in hsCRP in responders	More effective when baseline hsCRP > 5 mg/dL
Webers, 2020 [53]	Infliximab (infusions of 5 mg/kg infliximab or placebo at weeks 0, 2, 6, 12, and 18; from week 24 until week 54, all patients received infliximab therapy)	23 patients with ankylosing spondylitis	54 weeks	CRP levels not related to depression	Effective in improving symptoms
Loftus, 2008 [57]	Adalimumab, Induction: open-label adalimumab 80-mg, then a 40-mg dose at week 2; then adalimumab 40 mg weekly/every other week or placebo injections	499 patients with Crohn’s disease	56 weeks	Not measured	Effective; no difference between adalimumab weekly/every other week for all visits
Menter, 2010 [58]	Adalimumab, 40 mg every other week	96 patients with psoriasis	12 weeks	Not measured	Effective
Scheinfeld, 2016 [55]	Adalimumab, 40 mg weekly/every other week	154 patients with hidradenitis suppurativa	16 weeks	Not measured	Effective in patients with high baseline pain
Simpson, 2016 [59]	Dupilumab, 100 mg every 4 weeks/200 mg every 2 weeks/300 mg every 2 weeks/300 mg QW	380 adults with moderate to severe atopic dermatitis	16 weeks	Not measured	Effective (300 mg weekly/every 2 weeks)
de Bruin-Weller, 2018 [60]	Dupilumab, 300 mg weekly/every 2 weeks + topical corticosteroids	318 adults with atopic dermatitis	16 weeks	Not measured	Effective
Cork, 2020(SOLO 1 and 2) [61]	Dupilumab, 300 mg weekly/every 2 weeks	1379 patients with atopic dermatitis for ≥ 3 years	16 weeks	Not measured	Effective
Tyring, 2006 [54]	Etanercept, 50 mg BIW	618 patients with psoriasis	12 weeks	Not measured	Effective
Tyring, 2013 [56]	Etanercept; Group A: etanercept 50 mg BIW for 12 weeks, followed by etanercept 50 mg QW and placebo QW for 12 weeks. Group B: placebo BIW for 12 weeks, followed by etanercept 50 mg BIW for 12 weeks.	121 patients with moderate-to-severe plaque psoriasis with scalp involvement	24 weeks	Not measured	Effective
Langley, 2010 [64]	Ustekinumab, 45 or 90 mg at weeks 0, 4, and every 12 weeks through week 52, or placebo at weeks 0 and 4 + 45 or 90 mg of ustekinumab at weeks 12, 16, and every 12 weeks	1230 patients with psoriasis	Results reported through 24 weeks	Not measured	Effective
Griffiths, 2017 [62]	Ixekizumab, 80 mg every 2/4 weeks, initial dose 160 mg; etanercept, 50 mg BIW	575 patients with psoriasis	12 weeks	Reduction in hsCRP	Ixekizumab effective
Gordon, 2018 [63]	Guselkumab, 100 mg at weeks 0, 4, 12, and 20; placebo at weeks 0, 4, 12 followed by guselkumab 100 mg at weeks 16, 20; or adalimumab, 80 mg at week 0, 40 mg at week 1, and 40 mg every-2-weeks through week 23	989 patients with psoriasis	24 weeks	Not measured	Guselkumab more effective

hsCRP, high sensitive C reactive protein; MDD, major depressive disorder; CRP, C reactive protein; BIW, twice weekly; QW, once weekly.

### 3.3. Corticosteroids

Other powerful anti-inflammatory drugs, corticosteroids, have generated positive results in unipolar depression, but their serious side effects represent an important disadvantage [47]. Moreover, there is a putative correlation between this type of medication and atypical depressive syndromes [65].

Regarding evidence in favour of an antidepressant role of corticosteroids, a 4-day course of treatment with dexamethasone in MDD patients was superior to placebo, based on HAM-D scores 14 days after the beginning of the intervention [66]. In turn, hydrocortisone produced an acute antidepressant effect compared to placebo and corticotropin-releasing hormone (CRH) in a double-blind, placebo-controlled study [67]. However, data from an RCT on cardiac surgery patients (*n* = 1244) revealed that a single intraoperative intravenous dose of dexamethasone does not impact depression, except for the female subgroup, which might be more affected by hypothalamic-pituitary-adrenal axis dysfunctions [68]. Moreover, in men with chronic pelvic pain syndrome, one month of treatment with oral prednisolone only led to a trend towards improving depression (quantified by Hospital Anxiety and Depression Scale), in the context of normal baseline values for these scores [69]. In patients with cancer-related fatigue, dexamethasone improved quality of life but did not exert an antidepressant effect [70].

Eventually, the results of a network meta-analysis imply that corticosteroids have a greater antidepressant capacity than other anti-inflammatory agents, but the head-to-head comparisons identified no statistically significant difference between these classes [71].

## 4. Non-Conventional Anti-Inflammatory Drugs

In addition to medications with a clear anti-inflammatory principal mechanism, several categories of drugs proved this type of effect in addition to their main activity.

### 4.1. Statins

Statins, besides their lipid-lowering properties, can modulate numerous components of both innate and adaptive immunity. Their main mechanism of action is the inhibition of the rate-limiting enzyme involved in cholesterol synthesis. However, it appears that statins exert an anti-inflammatory effect both indirectly, through the reduction of low-density lipoprotein, and directly, through the downregulation of CRP, cytokines and T helper cell activity [72]. One possible mechanism is the inhibition of nuclear factor κB (NF-κB)—a key regulator of pro-inflammatory cytokines [73]. These results and many more offer the rationale for studying the effect of statins on the inflammatory mechanism of unipolar depression.

Statins were mainly investigated as add-ons and, unlike NSAIDs, might be rather recommended to patients with an elevated cardiovascular risk [47]. At the one-year follow-up, in depressive patients with acute coronary syndrome, a double-blind, placebo-controlled trial of escitalopram revealed a reduction of depression scores caused by statin use. However, a naturalistic prospective observational cohort study found this response only associated with the lipophilic statins [74]. In post-coronary artery bypass graft surgery patients, the administration of simvastatin (20 mg/day) or atorvastatin (20 mg/day) for 6 weeks led to superior results for simvastatin regarding depression scores reduction, latency and response rate. Treatment adverse effects were not significant [75].

Simvastatin (20 mg/day) was also effective as an add-on to fluoxetine in an RCT on MDD patients [76]. In another trial, fluoxetine was supplemented with lovastatin (30 mg/day), generating, similarly, a positive response [77]. Atorvastatin (20 mg/day) was, too, investigated as an adjunctive to citalopram, and improved the symptomatology in MDD patients, although remission was not seen in any participant [78]. In contrast, a different study found no advantage for rosuvastatin as an adjunctive to antidepressant therapy in young MDD patients [33].

Overall, results from most meta-analyses and systematic reviews indicate a positive role of statins in MDD [79,80,81], even if monotherapy did not prove to be successful [82]. Another finding was related to lipophilic statins, especially atorvastatin and simvastatin, which seem to be superior to other drugs in this group, probably due to their enhanced capacity to cross the blood-brain barrier and exert their actions in the CNS [83,84].

### 4.2. Minocycline

Minocycline, a highly liposoluble tetracycline that can cross the blood-brain barrier, was also shown to possess anti-inflammatory actions. These might be explained by its antioxidant properties and its capacity to inhibit immune cell activity, apoptosis, and various inflammation-promoting enzymes [85]. Moreover, it inhibits specific pathways involved in the inflammatory mechanism of depression, such as the kynurenine and the p-38 pathways [86]. Minocycline’s antidepressant effect is also associated with the inhibition of microglial activation, strongly linked to neuroinflammation [87]. Together, these findings support the use of minocycline in unipolar depression.

Like previously discussed drugs, it was most frequently studied as an augmentation therapy. In a multi-site pilot RCT, minocycline (100 mg/day for 2 weeks, then 200 mg/day up to 12 weeks) added to the treatment as usual significantly ameliorated treatment-resistant MDD [88]. In contrast, also in treatment-resistant depressive patients, minocycline (200 mg/day for 4 weeks) was effective only at higher serum CRP baseline values, with a threshold of 2.8 mg/L. In addition, higher IL-6 predicted better response, and interferon (IFN)γ was significantly lowered after the intervention, further supporting an anti-inflammatory role of minocycline in alleviating this disease [89]. There is also an RCT on MDD patients (*n* = 71) that failed to prove a reduction in Montgomery-Åsberg Depression Rating Scale (MADRS) by minocycline (200 mg/day, 12 weeks) added to the usual treatment, although significant beneficial changes occurred in several clinical and quality of life scores [90].

Another subgroup that may benefit from this medication is represented by human immunodeficiency virus (HIV) patients with co-existing depression, as indicated by a study that assessed both its efficacy and safety [91].

Two meta-analyses found promising results for minocycline in unipolar depression, with the drawback of including only a small number of RCTs (3 and 4, respectively) [92,93].

### 4.3. N-Acetyl Cysteine (NAC)

Various nutraceuticals have also led to positive outcomes in unipolar depression. One example is NAC—most commonly used to counteract acetaminophen toxicity and in dysfunctions of mucus secretion, such as cystic fibrosis or pneumonia [94]. In addition, it possesses numerous systemic roles. Thus, as part of its anti-oxidant effect, NAC has the role of restoring cellular levels of glutathione, a critical non-enzymatic cerebral anti-oxidant, and acts as a scavenger of reactive oxygen species [95]. NAC also exerts an anti-inflammatory role by decreasing various cytokines in the brain, especially IL-6, TNF-α and IL-1β, which are involved in the inflammatory mechanisms of depression [10,96]. These effects made NAC be of interest in the treatment of MDD.

A particular use for NAC, which needs further investigation, might be in suicidal patients. To this end, a recent naturalistic study on patients suffering from a major depressive episode who had ingested an intentional overdose of different medications concluded that, after one week, there was a significant reduction in suicidality and depressive symptoms in participants who received NAC (for acetaminophen) compared to those on other supportive treatments for other overdoses [97].

A pattern similar to minocycline was observed in an RCT, where adjunctive NAC (1.8 g/day, 12 weeks) improved depressive symptoms only conditioned by baseline hsCRP > 3 mg/L. Additionally, hsCRP was significantly more reduced throughout the study in the intervention group [98]. NAC (2 g/day) was also studied as an add-on to the usual treatment of MDD patients, generating positive outcomes, but, this time, there were no significant changes in IL-6, CRP or BDNF concerning this medication [99]. Another RCT with NAC as adjunctive found an improvement in response and remission rates only at week 16 (after discontinuation), counterbalanced by an increase in gastrointestinal and musculoskeletal adverse events [100]. These results show that NAC might be a suitable adjuvant medication in unipolar depression, especially in patients displaying a pro-inflammatory status. In addition, it might represent a therapeutic option for suicidal patients. However, future studies are warranted to establish its efficacy and safety profile.

### 4.4. Omega-3 Polyunsaturated Fatty Acids (n-3 PUFAs)

Omega-3 polyunsaturated fatty acids represent another novel promising intervention in MDD, with the advantage of simultaneously acting on multiple pathways pertaining to inflammation and oxidative and nitrosative stress [101]. Their use in this pathology is also justified by the association between low blood or erythrocyte n-3 PUFAs levels and depressive symptoms [102,103]. It has been proposed that the anti-inflammatory action of n-3 PUFAs is mediated by their metabolites, namely resolvins, maresins and protectins [104].

As a summary of the existing RCTs’ outcomes, omega-3 fatty acids seem to exert their antidepressant actions especially in individuals with already established MDD [105,106,107,108,109,110,111] and without comorbid anxiety disorders [112]. A summary description of RCTs of omega-3 fatty acids in patients with MDD can be found in Table 3. On the contrary, several RCTs reported opposite effects and, as a result, the role of omega-3 fatty acids in the treatment of MDD is still under debate and further studies are needed to provide conclusive statements [107,113,114,115,116].

Some studies explored the potential depression preventive effects of omega-3 fatty acids. With respect to this role, omega-3 fatty acids are less efficacious in healthy subjects [117,118], women at risk for peripartum depression [119,120], or patients with high cardiovascular risk [121,122]. Interestingly, Su et al. proved that n-3 PUFAs had a therapeutic benefit in pregnant women suffering from MDD and displayed no adverse effects on patients or newborns. These results are of most importance for the development of different treatment options since MDD during pregnancy adversely affects the mother and the child and current pharmacological treatment (i.e., SSRI and SNRI) may determine several negative consequences for the offspring [123,124]. However, for patients with perinatal MDD, n-3 PUFAs do not seem to have the same positive effect as for patients with MDD during pregnancy [125,126].

Even though their outcomes are not generally important in size, they are effective in patients with low-grade inflammation and it was observed that a higher eicosapentaenoic acid (EPA) content and the add-on type of treatment may predict a more successful response [127]. As previously discussed in the case of cytokine inhibitors or minocycline, a study on MDD patients concluded not only that EPA is more effective in those who display pre-existent inflammation, but it is also inferior to placebo in those who do not [110].

Unfortunately, several studies that assessed the role of omega-3 fatty acids in patients with cardiovascular diseases or diabetes mellitus and comorbid unipolar depression failed to show an effective role of this pharmacological intervention [128,129,130,131,132]. Jiang et al. reported positive results only on cognitive symptoms of depression and social functioning in patients with chronic heart failure and MDD who underwent treatment with omega-3 fatty acids [130]. On the other hand, an RCT showed that n-3 PUFAs yielded a beneficial effect in patients with obesity or overweight and MDD [133]. Moreover, these results outline the importance of the intervention group selection, i.e., patients with low-grade inflammation, since adipose tissue is known as a source of cytokines in the inflammatory mechanism of unipolar depression [10].

Encouraging results have been reported in the younger population. Thus, a study found that EPA + docosahexaenoic acid (DHA) effectively reduced depression scores in children—a subgroup where optimal therapies are less defined [134], and the same combination induced an important and relatively fast remission of symptomatology in young students [135]. Interestingly, omega-3 supplementation also managed to relieve depressive symptoms in adolescents with borderline personality disorder, a particular situation where there are fewer treatment options available [136]. Thus, despite the conflicting results obtained so far, n-3 PUFAs might be appropriate for treating unipolar depression in several specific categories, i.e., MDD patients, pregnant women, obese patients and younger individuals.

**Table 3:** Summary of randomised controlled trials of omega-3 fatty acids in MDD.

Study	Intervention and Treatment Regimen	Participants	Duration of Intervention	Immune Parameters	Main Outcomes
MDD patients without comorbidities					
Nemets, 2002 [105]	E-EPA (derived from 96% pure fish oil), 2 × 1 g/day (add-on to treatment as usual)	20 patients with current MDD	4 weeks	Not measured	Effective
Marangell, 2003 [113]	DHA, 2 g/day (monotherapy)	36 patients with MDD	6 weeks	Not measured	Not effective
Su, 2003 [106]	EPA 440 mg + DHA 220 mg/capsule, 10 capsules/day(add-on to treatment as usual)	28 patients with MDD	8 weeks	Not measured	Effective
Silvers, 2005 [114]	3 g of omega-3 PUFAs (0.6 g EPA, 2.4 g DHA) per day(add-on to treatment as usual)	77 participants being treated for a current depressive episode	12 weeks	Not measured	Not effective
Grenyer, 2007 [115]	3 g of omega-3 PUFAs (2.2 g DHA, 0.6 g EPA)/day (monotherapy or add-on to treatment as usual)	83 patients with MDD	4 months	Not measured	Not effective
Jazayeri, 2008 [107]; Jazayeri, 2010 [137]	EPA (1000 mg/day)/fluoxetine (20 mg/day)/combination	60 patients with MDD	8 weeks	Serum cortisol decreased in all 3 groups, IL-1β and IL-6 not changed	Both equally effective, combination superior
Lespérance, 2011 [112]	1050 mg/day of EPA and 150 mg/day of DHA (monotherapy or add-on to treatment as usual)	432 patients with MDD, clinically significant symptoms ≥ 4 weeks	8 weeks	Not measured	Effective in patients without comorbid anxiety disorders
Rondanelli, 2012 [108]; Rizzo, 2012 [138]	1.67 g EPA + 0.83 g DHA/day (monotherapy)	46 elderly female patients with MDD or dysthymia, 66–95 years old	8 weeks	CD2, CD3, CD4, CD8, CD19 lymphocytes, IL-5, IL-15—not significantly changed; increase in CD16 lymphocytes with a possible role in inflammation	Effective
Gertsik, 2012 [109]	900 mg EPA + 200 mg DHA + 100 mg other omega-3 fatty acids/day (add-on to citalopram 20/40 mg/day)	42 patients with MDD	8 weeks	No changes in plasma CRP	Effective as add-on
Mischoulon, 2015 [116]	EPA-enriched n-3 1000 mg/day/DHA-enriched n-3 1000 mg/day (monotherapy)	154 adults with MDD	8 weeks	Not measured	Not effective
Rapaport, 2016 [110]	2 capsules/day of EPA-enriched mix (ProEPAxtra, 530 mg EPA/130 mg DHA per soft gel) and 2 placebo capsules/4 capsules/day of DHA-enriched mix (ProDHA, 225 mg DHA/45 mg EPA per soft gel) (monotherapy)	155 patients with MDD	8 weeks	High IL-1ra or hs-CRP or low adiponectin associated with better response to EPA; high hsCRP, IL-6 or leptin associated with worse response to placebo	EPA more effective and DHA less effective with high baseline inflammation markers; EPA inferior to placebo and DHA with low baseline inflammation
Su, 2018 [111]	EPA (3.5 g/day) or DHA (1.75 g/day) (monotherapy)	27 patients with MDD	12 weeks	cPLA2 expression increased in EPA group, tendency for decrease in COX-2 expression in both groups	Both effective
Jahangard, 2018 [139]	1000 mg/day omega-3 PUFA (add-on to sertraline 50–200 mg/day)	50 outpatients with MDD	12 weeks	Not measured	Effective
Patients with perinatal MDD					
Freeman, 2008 [125]	1.1 g of EPA + 0.8 g of DHA (+psychotherapy for all subjects) (monotherapy)	59 women with perinatal MDD, no antidepressant treatment at the moment of the study	8 weeks	Not measured	Not effective
Rees, 2008 [126]	6 g/day fish oil (27.3% DHA, 6.9% EPA and 3.3% omega-6 fatty acids) (monotherapy)	26 women with perinatal MDD, no antidepressant treatment at the moment of the study	6 weeks	Not measured	Not effective
Su, 2008 [140]	3.4 g omega-3 PUFAs/day (monotherapy)	36 pregnant women with MDD	8 weeks	Not measured	Effective
MDD patients with comorbidities					
Carney, 2009 [128]; Bot, 2011 [141]	930 mg of EPA and 750 mg of DHA/day (add-on to sertraline 50 mg/day)	122 patients with coronary heart disease and MDD	10 weeks	Baseline hs-CRP, IL-6, and TNF-α not associated with symptoms, response or remission after sertraline treatment; no influence of omega-3	Not effective
Bot, 2010 [129]; Mocking, 2012 [142]	E-EPA (1 g/day) (add-on to treatment as usual)	25 adults with diabetes mellitus and MDD, with antidepressant treatment at the moment of the study	12 weeks	Serum CRP, IL-6 and TNF-α not changed	Not effective
Keshavarz, 2018 [133]	6 capsules/day, containing 180 mg EPA, and 120 mg DHA each (monotherapy)	65 women with overweight/obesity and MDD	12 weeks	Not measured	Effective
Jiang, 2018 [130]	4 capsules of 400/200 EPA/DHA 500 mg per capsule (“2:1 EPA/DHA”), 4 capsules of almost pure EPA 500 mg per capsule (“high EPA”), or 4 capsules of corn oil (“placebo”), daily (monotherapy or add-on to treatment as usual)	108 patients with chronic heart failure and MDD	12 weeks	Not measured	Effective only on cognitive depressive symptoms and social functioning
Carney, 2019 [131]	EPA 2 g/day (as add-on to sertraline 50 mg/day)	144 patients with/at high risk for coronary heart disease and MDD	10 weeks	Not measured	Not effective as add-on
Chang, 2020 [132]	2 g EPA + 1 g DHA/day (monotherapy)	59 patients with cardiovascular diseases and MDD	12 weeks	Not measured	Not effective, but improved core depression symptoms in the very severe MDD group

MDD, major depression disorder; EPA, eicosapentaenoic acid; DHA, docosahexaenoic acid; PUFA, polyunsaturated fatty acids; IL, interleukin; CD, cluster of differentiation; COX, cyclooxygenase; hsCRP, high sensitive C reactive protein; CRP, C reactive protein; PLA2, phospholipase A2; TNF-α, tumor necrosis factor-alpha.

### 4.5. Probiotics

Probiotics have begun to be considered as antidepressant agents, as it was shown that an imbalanced gut microbiota with an increase in pro-inflammatory species is an important component of MDD [9]. In this regard, a recent systematic review on clinical anxiety and depression found a higher prevalence of *Enterobacterales*, *Enterobacteriaceae*, *Eggerthella*, *Desulfovibrio*, which are associated with gastrointestinal inflammation. There was also an underrepresentation of *Faecalibacterium*, *Coprococcus*, *Clostridium* XIVa—species which produce short-chain fatty acids with an anti-inflammatory role [143]. As a general explanation, several bacterial compounds, such as lipopolysaccharide, bacterial lipoprotein, flagellin, and CpG DNA, can generate an increase in cytokines through the activation of the immune system [144]. This microbiota dysregulation-induced peripheral inflammation (with implications on the CNS, as previously discussed) is further augmented by the increase in intestinal permeability and ensuing bacterial translocation encountered in this condition [145].

Conversely, probiotics were proved to exert an anti-inflammatory effect not only in the intestine, but at a more general level, as they can reduce systemic pro-inflammatory cytokines such as IL-1β, TNF-α, IL-6, and IFN-γ [146]. Stimulation of IL-10 and regulatory T cell population by the probiotic microorganisms appears to be one of the mechanisms involved [147].

A number of RCTs have shown, consequently, positive effects of probiotics in MDD patients [148,149]. A summary description of existing RCTs of probiotics in patients with MDD can be found in Table 4. Recently, Schaub et al. have proved the efficacy of a short course of treatment with high-dose probiotics in depressive patients, also supported by changes in the microbiota (elevated levels of *Lactobacillus*, correlated with the improvement in symptomatology) and neuroimaging parameters [150]. Several studies failed to demonstrate a clinical improvement following probiotics administration [151,152,153]. Nevertheless, they reported an enhancement in cognitive function [151] and inflammatory biomarkers [152,153]. Given the diversity of utilised strains and doses, there is a clear need for further studies to establish the optimal therapeutic choice.

In contrast to omega-3 fatty acids, probiotics also seem to exert antidepressant actions in healthy subjects [154,155]. Other categories that might benefit from this intervention according to trials are patients with plaque psoriasis or multiple sclerosis—diseases with an important inflammatory component [156,157].

Several meta-analyses were conducted, generating significant results, which were commonly more prominent in clinically depressed individuals than in healthy ones [158,159,160,161], suggesting a more important role of probiotics in treating than preventing MDD. It was also argued that multiple strains are more powerful than single strains [161].

**Table 4:** Summary of randomised controlled trials of probiotics in MDD.

Study	Intervention and Treatment Regimen	Participants	Duration of Intervention	Immune Parameters	Main Outcomes
Akkasheh, 2016 [148]	Lactobacillus acidophilus, Lactobacillus casei, Bifidobacterium bifidum, each 2 × 109 CFU/g, daily (monotherapy)	40 patients with MDD, 20–55 years old	8 weeks	Decreased hsCRP	Effective
Kazemi, 2019 [162]	Lactobacillus helveticus and Bifidobacterium longum (≥10 × 109 CFU per 5 g sachet)/prebiotic (galactooligosaccharide) (monotherapy)	81 patients with MDD	8 weeks	kynurenine/ tryptophan ratio decreased, tryptophan/ isoleucine ratio increased	Probiotic effective, prebiotic not effective
Rudzki, 2019 [151]	10 × 109 CFU of probiotic bacteria Lactobacillus Plantarum 299 v/capsule, 2 capsules/day(add-on to an SSRI)	79 patients with MDD	8 weeks	Decrease in kynurenine, increase in 3-hydroxykynurenine: kynurenine; TNF-α, IL-6 and IL-1b—not changed	Not effective, but improvement in cognitive function
Reininghaus, 2020 [152]; Reiter, 2020 [163]	OMNi-BiOTiC^®^ Stress Repair (3 g/day, 7.5 × 109 CFU, Lactobacillus casei W56, Lactobacillus acidophilus W22, Lactobacillus paracasei W20, Bifidobacterium lactis W51, Lactobacillus salivarius W24, Lactococcus lactis W19, Bifidobacterium lactis W52, Lactobacillus plantarum W62, Bifidobacterium bifidum W23) + 125 mg biotin (add-on to treatment as usual)	61 inpatients with MDD	28 days	Upregulation of IL-17 pathways; reduction in IL-6 gene expression; no change in TNF and NF-κB	Not effective (regarding clinical parameters)
Zhang, 2021 [153]	100 mL of a Lacticaseibacillus paracasei strain Shirota beverage (108 CFU/mL) (monotherapy)	82 patients with constipation and MDD	9 weeks	Reduction in IL-6	Not effective
Tian, 2022 [149]	Bifidobacterium breve CCFM1025 1010 CFU/day (add-on to treatment as usual)	45 patients with MDD	4 weeks	No change in serum TNF-α and IL-β	Effective
Schaub, 2022 [150]	Vivomixx^®^ (Streptococcus thermophilus NCIMB 30438, Bifidobacterium breve NCIMB 30441, Bifidobacterium longum NCIMB 30435—Re-classified as Bifidobacterium. lactis, Bifidobacterium infantis NCIMB 30436—Re-classified as B. lactis, Lactobacillus acidophilus NCIMB 30442, Lactobacillus plantarum NCIMB 30437, Lactobacillus paracasei NCIMB 30439, Lactobacillus delbrueckii subsp. Bulgaricus NCIMB 30440—Re-classified as Lactobacillus helveticus), 900 billion CFU/day (add-on to treatment as usual)	47 patients with MDD	31 days	Not measured	Effective

CFU = colony-forming units; MDD, major depression disorder; hsCRP, high sensitive C reactive protein; TNF, tumor necrosis factor; IL, interleukin; NF-κB, nuclear factor kappa-light-chain-enhancer of activated B cell.

## 5. Physical Exercise

Physical exercise is not just an addition of minor importance to depression therapy, as it possesses several well-documented effects on its pathophysiology. It can interfere with the kynurenine pathway (a key factor in inflammatory depression [25]) by transforming this compound into kynurenic acid—unable to cross the blood-brain-barrier [164]. Moreover, it can reduce TNF-α and IL-1α production, the number of several monocyte subtypes together with toll-like receptor (TLR) 4 and lipopolysasscharides (LPS) expression, and macrophage accumulation in adipose tissue, and activate the anti-inflammatory cholinergic component [165].

In a clinical setting, akin to the previously mentioned pharmacological interventions, it seems beneficial to patients with comorbidities. A study on 80 participants suffering from chronic obstructive pulmonary disease found that aerobic exercise decreased both plasma cytokines and depressive symptoms [165]. In an RCT on diabetes mellitus type 2 patients, physical exercise ameliorated inflammation, oxidative damage and subsyndromal depression to the same extent as psychoeducation and diabetes mellitus reeducation [166]. In gastro-esophageal junction cancer patients, physical training alleviated depressive symptoms, while preventing the inflammation-induced conversion of kynurenine to neurotoxic metabolites [167]. Finally, a study on multiple sclerosis patients reported that exercise reduced the IL-2 level in the cerebrospinal fluid together with depressive symptoms [168].

A randomised trial on MDD patients revealed that, similar to anti-inflammatory pharmacological interventions, the effectiveness of exercise was predicted by higher baseline levels of TNF-α, and IL-1β reduction was associated with an enhancement of symptoms [169]. Also, exercise in addition to cognitive-behavioural therapy increased the anti-inflammatory cytokine IL-10 and reduced CRP in the subgroup with higher CRP levels [170]. Moreover, a higher baseline IL-6 level, as well as its reduction, were significantly correlated with a decrease in unipolar depression severity following 12 weeks of physical exercise [171]. Taken together, these findings reveal a strong connection between the inflammatory status of patients and the efficacy of exercise intervention in unipolar depression.

Another key aspect is the type or the intensity of exercise. For instance, in older adults, aerobic exercise reduced serum IL-6, IL-18 and CRP compared to flexibility/moderate strength training [172]. However, a study on university students concluded that high-intensity exercise might be detrimental, as it increases TNF-α, IL-6 and perceived stress. In contrast, moderate-intensity exercise had positive outcomes on both inflammatory cytokines and depressive symptoms [173].

## 6. Electroconvulsive Therapy (ECT)

ECT, a major option especially in treatment-resistant MDD [174], has also been shown to influence inflammation in this pathology. While the acute reaction to this therapy engenders a transient rise in inflammation, there seems to be a subsequent decrease in cortisol and several cytokines [175]. The latter might possess clinical significance since a strong connection was found between ECT-induced IL-6 reduction and hippocampal volume increase on magnetic resonance imaging [176]. Even a single ECT session exerts an inhibitory effect on several inflammatory pathways, such as NF-κB and inducible nitric oxide synthase [177].

The effect of this therapy in treatment-resistant unipolar depression seems to be, to a certain extent, mediated by its anti-inflammatory properties. Thus, response and remission were associated with a decrease in cerebrospinal fluid levels of IL-17, macrophage inflammatory protein-1α, Rantes and IL-2R. Conversely, the same compounds increased in non-responders/non-remitters during ECT. However, the same study reported an overall increase in pro-inflammatory markers in the cerebrospinal fluid during the first week after the last ECT session, suggesting a more complex action of this intervention [178]. Similarly, the decrease in serum IL-6, a key inflammatory cytokine, was associated with remission [179]. This finding might be of particular importance since IL-6 is considered a potential biomarker for treatment-resistant unipolar depression [180]. Another compound that decreases following ECT only in remitters is the macrophage migration inhibitory factor, which promotes the generation of inflammatory cytokines [181]. Decreases in cytokine levels induced by ECT were also linked to the treatment response (TNF-α) [182] and the disease severity attenuation (IL-5) [183].

In contrast, several studies did not find significant alterations in cytokine levels [184,185], while other ones identified changes that were not correlated with the clinical response [186]. It should be taken into account that the characteristics of the participants varied across the studies (e.g., melancholic subtype of unipolar depression, presence of psychotic symptoms), that different stimulation protocols were utilised and that cytokine levels were assessed at various time points in relation to the therapy. Moreover, sex-related differences should be further investigated, since recent data indicate an association between IL-8 levels and treatment outcomes only in female participants [187].

A systematic review identified no consistent changes in particular biomarkers in relation to ECT, but TNF-α and IL-6 were more frequently lowered, and IL-10 (anti-inflammatory cytokine) and IL-8—augmented after treatment, supporting its proposed role in modulating the immune system, even in the context of underpowered studies [188].

As previously seen in anti-inflammatory therapies, response to ECT is predicted by higher baseline biomarkers, such as IFN-γ-induced protein 1, macrophage inflammatory protein-1β, IL-2R [178], CRP (in elderly) [189] and IL-6 and CRP in women [190].

A serious concern regarding this intervention is related to cognitive impairment in older patients, which was found to be greater when baseline CRP and cytokine levels were higher, in a prospective cohort study [191].

## 7. Psychological Therapy

Several studies have already shown a putative influence of psychotherapy on inflammation, suggesting an unexpected potential role for this therapeutic option. Moreover, this anti-inflammatory effect appears to be linked with the clinical efficacy of this type of intervention in some studies. For instance, an RCT revealed that psychotherapy simultaneously decreased depression scores and IL-6 and TNFα values, which were correlated with the social role component of one score [192]. In another RCT, seven weekly sessions of cognitive behaviour treatment (CBT) or narrative therapy both decreased symptomatology, but only the former induced a reduction of TNF-α and IL-6 and was also more effective from a clinical point of view—although these effects were not correlated [193]. Interestingly, CBT, but not supportive psychotherapy, downregulated microglial activation in association with symptomatology, which further supports the efficacy of this type of therapy [194]. In patients with clinical unipolar depression, CBT also normalised the level of numerous cytokines [195]. Furthermore, it downregulated the inflammatory TLR4-NFκB pathway, possibly activated by bacterial translocation, and this change was also correlated with clinical enhancement [196]. More recently, it was suggested that psychotherapy also modulates inflammation in depression at the epigenetic level [197].

In contrast, a study that assessed depression and anxiety found that mindfulness or CBT did not elicit any change in inflammatory markers, but in epidermal growth factor- which was also associated with treatment response, suggesting other possible mechanisms involved [198]. Some studies yielded negative results regarding the influence of psychotherapy on inflammation, or did not find a decrease in inflammation despite the presence of symptom improvement [199].

Regarding baseline inflammation, two studies concluded that it predicted worse treatment outcomes [200,201]. However, a recent study indicates that patients with peripheral blood mononuclear cells producing higher TNF-α levels might benefit more from multimodal psychotherapy. Thus, this type of therapy appears efficacious in a subtype of patients at risk of later developing a pro-inflammatory state [202].

A meta-analysis concluded that psychological interventions can reduce pro-inflammatory biomarkers (although the effect size was small), and significant findings were obtained especially regarding CRP [203]. A systematic review focused on CBT stated that, in most studies investigating a relationship between this type of intervention and inflammatory markers, a reduction was identified in the latter [204].

## 8. Conclusions

Undoubtedly, the connection between inflammation and unipolar depression outlined by recent studies is a promising avenue for future research. Collectively, the results presented by this review reinforce the need for investigating the exact significance of inflammation with regard to this complex pathology.

The clear benefit of the anti-inflammatory treatment in unipolar depression is supported by the positive outcomes of numerous RCTs. In some cases, the involvement of this particular mechanism in the antidepressant effect was suggested by the correlation between clinical improvement and changes in various biomarkers. Importantly, the pre-existing pro-inflammatory status was, in the vast majority of cases, a predictor of clinical efficacy. Apart from that, the studies under discussion revealed that each class might be suitable for specific types of patients. NSAIDs appear to be beneficial in MDD with or without comorbidities such as osteoarthritis, cancer or brucellosis, but not in healthy individuals. Given their most common uses, cytokine inhibitors were studied the most in immunological disorders such as Chron’s disease or psoriasis, but also in treatment-resistant unipolar depression with increased inflammation, where they were shown to be effective. Corticosteroids, despite issues regarding side effects, could improve outcome in several situations, especially in MDD and the female subgroup.

It is of note the inclusion, in this review, of several drugs and non-pharmacological interventions with recently discovered anti-inflammatory properties. These findings promote a better understanding of their mechanisms of action and possible clinical applications, but also the introduction of a larger array of therapies in the study of unipolar depression. For instance, statins, originally used in high cardiovascular risk individuals, proved to exert an antidepressant action in this condition, but also in MDD patients, as an add-on. The antibiotic minocycline was successful as an adjunctive in MDD with treatment resistance or coexisting HIV infection. Nutraceuticals represent another option that should be further explored. Thus, NAC produced positive results in MDD presenting with high inflammation and in suicidal patients, but its safety warrants future studies. Omega-3 fatty acids alleviated depressive symptoms especially in clinical MDD, pregnancy, obesity and the younger population, but did not exert a significant preventive role. In contrast, probiotics were also effective in healthy participants, but their contribution was more substantial in MDD, with or without comorbidities. Finally, physical exercise and ECT might also be appropriate for depressive patients with elevated inflammatory markers. The existing results do not support a similar role for psychological therapy, but a tendency to develop chronic inflammation might also predict treatment success.

Considering all these data, it can be argued that treatments with anti-inflammatory action have the potential to enhance clinical outcomes in unipolar depression, at least for some categories of patients. Thus, there is an urgent need for more studies establishing the optimal treatment regimens and the precise features of the individuals who could benefit most from this type of intervention. Finally, these results should be integrated in clinical practice, as a step forward towards personalised medicine.

## 9. Current Limitations and Future Research Directions

As highlighted above, the use of anti-inflammatory interventions in unipolar depression is still restricted by the limited current understanding of the pathophysiology involved. However, the lack of positive results in some of the existing studies might be explained by technical issues. For example, they assessed insufficient or no biomarkers, or did not stratify response and/or remission according to the inflammatory status. The determination of cut-off biomarker values could be an effective modality to distinguish between responders and non-responders. Moreover, the choice of population is crucial since, according to existing results, a positive response is more likely in MDD patients with inflammation or inflammation-related comorbidities than in healthy participants or MDD patients with normal immunological parameters. The dose and the treatment duration can also exert a great impact on the treatment success as well as the type of regimen (add-on or monotherapy). In addition, more future studies that will explore these interventions in combination with specific add-ons rather than treatment as usual would shed more light on the exact possible efficacious treatment regimens that could be used in clinical practice. Finally, the study design and the sample size can influence study outcome. Thus, there is an urgent need for more RCTs investigating the interventions under discussion, as they provide the most accurate measurement of treatment efficacy.

Future research should aim at a more detailed understanding of the inflammatory pathways involved in this pathology. This would enable a much more targeted intervention and a reduction in side effects. To this end, a multi-omics and systems biomedicine approach has been suggested [101]. Additionally, a more accurate definition of inflammatory MDD should be provided, possibly taking into account the association between symptomatology and specific aspects of the inflammatory response. Thus, particular subcategories of patients that could benefit from this type of interventions could be identified. For instance, additional studies are needed in suicidal patients since they have been shown to exhibit increases in inflammatory cytokines, which are considered promising biomarkers in addition to BDNF, hypothalamic-pituitary-adrenal axis activity, lipid profile and neuromodulators [205,206,207]. Furthermore, some components of inflammation, such as CRP, could be associated with distinct clinical pictures and might be considered as biomarkers for MDD [208].

Further studies should also focus on other medications with recently highlighted anti-inflammatory properties, which were not mentioned in this review. Such is the case of already classic antidepressants (i.e., SSRIs and SNRIs) or newer ones, such as agomelatine, which was shown to induce an important reduction in serum CRP levels [209]. Following the example of statins, minocycline or NAC, several drugs, such as ketamine [210], modafinil [46], oral antidiabetic drugs (i.e., pioglitazone and metformin) [211] or antiparkinsonian pramipexole [212], have also been re-purposed as possible antidepressant and anti-inflammatory agents in unipolar depression in some studies and definitely deserve future research. Finally, the results presented in this review might be further supported by the findings of ongoing studies on NAC, minocycline and simvastatin, for which protocols are available [213,214,215].

## Data Availability

Not applicable.
